# Users’ Motivations for Facebook Unfriending During the COVID-19 Pandemic: Survey Study

**DOI:** 10.2196/48908

**Published:** 2023-08-28

**Authors:** Stephen Neely

**Affiliations:** 1 School of Public Affairs University of South Florida Tampa, FL United States

**Keywords:** COVID-19, social media, unfriending, Facebook, survey, social networking, utility, accuracy, users, sex, age, race, ethnicity, political affiliation, survey study, web-based interaction, on the web

## Abstract

**Background:**

Social networking sites (SNSs) such as Facebook have been central to the global exchange of health-related information throughout the COVID-19 pandemic, but during this time, increased web-based interactions proved to be a source of stress and conflict for many SNS users. Prior research suggests that many users have engaged in significant *boundary regulation* during this period, using behaviors such as *unfriending* to refine and reorient their social networks in response to pandemic-related information.

**Objective:**

This study aimed to examine Facebook unfriending during and in relation to the first year of the pandemic to better understand how SNS users have managed and maintained their social networks around the COVID-19 pandemic. On the one hand, unfriending may be motivated by an attempt to protect the utility and accuracy of a user’s informational environment. On the other hand, it may be motivated by a desire to tune out alternative viewpoints and opinions. Both motivations may have significant implications for public health discourse and outcomes.

**Methods:**

A sample of 824 active Facebook users (drawn from a representative survey of 1000 American adults) was analyzed using a series of logit regression models. Survey respondents were selected using a stratified quota sampling approach to ensure a representative sample of the US population. Balanced quotas were determined (by the region of the country) for sex, age, race, ethnicity, and political affiliation.

**Results:**

In total, 31.7% (261/824) of active Facebook users unfriended at least one account over COVID-19 pandemic–related posts during the first year of the pandemic. The most common reasons for unfriending included “making political comments about COVID-19” (191/824, 23.2%) and “posting information that was inconsistent with public health guidelines” (162/824, 19.7%). As hypothesized, reliance on Facebook for COVID-19 pandemic–related news and information was associated with a greater likelihood of unfriending, particularly in response to information that was inconsistent with public health guidelines. Political factors (particularly partisan intensity) were also predictive of unfriending, especially in the case of COVID-19 pandemic–related disagreements.

**Conclusions:**

Both information utility concerns and political factors were associated with a greater likelihood of COVID-19 pandemic–related unfriending, although the magnitude of the effects associated with utility appears to be greater. Although utility-motivated unfriending may lead to more reliable health information experiences for some SNS users, the tendency of consumers to assess accuracy and credibility on the basis of partisan predilections obscures this finding and warrants further consideration.

## Introduction

### Overview

Throughout the COVID-19 pandemic, social networking sites (SNSs) such as Facebook and Twitter have been central to the global exchange of health-related information. SNS users around the world have relied on these sites to learn and stay informed about the evolving pandemic, whereas public health organizations have used the same platforms to promote public health and disease prevention guidelines. In the United States, more than three-quarters (76%) of SNS users reported having relied on social media at least “a little” to stay informed about the COVID-19 pandemic, whereas approximately half (46%) have relied on it "a lot" [1]. Similar trends have been noted among Chinese [2] and European [3] SNS users. Although social media’s growing role in the exchange of health information predates the COVID-19 pandemic [4,5], the past 2 years have undoubtedly seen a fundamental shift in the locus of health information seeking for millions of SNS users around the world.

On the one hand, the evolution of social media into a forum for public health discourse promises greater access and connectivity for both consumers and health care providers. On the other hand, the susceptibility of web-based social networks to misinformation and politicization has emerged as a significant source of concern over recent years [6-8]. The COVID-19 pandemic, and the accompanying *infodemic*, highlighted the magnitude of these concerns and their potential impacts on both personal and public health outcomes [9,10]. Data show that disagreements over the COVID-19 pandemic, which often center around misinformation and political constructions of the pandemic, have caused significant confusion for health consumers and placed a strain on interpersonal relationships and social networks [11-13]. During this time, many SNS users engaged in significant boundary regulation (or renegotiation of their social networking communities) through processes such as *following*, *blocking*, and *unfriending* others in response to COVID-19 pandemic–related content. Network theory suggests that these microlevel behaviors can have significant macrolevel impacts on the broader societal exchange of health-related information [14].

This study aimed to examine COVID-19 pandemic–related unfriending on Facebook during the first year of the pandemic. Survey data show relatively high levels of COVID-19 pandemic–related unfriending during this period [15], but understanding *why* individuals break network ties in the face of such disagreements is important. Are those who unfriend simply *cleaning up* their information environment to ensure its accuracy and utility or are they *tuning out* competing points of view? The answer is of significance to health professionals, public health officials, and communication scholars alike, as boundary regulation has significant implications for the functioning of social networks, including their informational credibility and openness to corrective information. Although previous studies have examined the frequency of and motivations for unfriending in sociopolitical contexts [16-18], relatively little attention has been paid to unfriending in the context of public health discourse.

If SNS users unfriend accounts as a way of tuning out competing public health viewpoints, there may be a significant and systemic impact on the exchange of accurate and corrective health information over the long run, as these decisions block one’s subsequent exposure to potentially valuable information from these sources. If, on the other hand, SNS users engage in unfriending as a way of safeguarding the accuracy and credibility of their informational environments, then these behaviors may have a net positive effect on public health outcomes. In this study, responses from a national survey of US-based adults (n=824) were examined to better understand (1) how prevalent COVID-19 pandemic–related unfriending was during the first year of the COVID-19 pandemic and (2) which factors motivated these boundary-regulating decisions. By answering these questions, we can better understand how SNS users have managed their social networks during the pandemic as well as how these decisions might influence their subsequent information exposure and health learning. The implications of this analysis are discussed in the context of recent literature, including the potential costs and benefits of health-related unfriending.

### Background Literature

To date, academic analyses of unfriending on social media have focused primarily on sociopolitical contexts such as election cycles [16], protest movements [17], and geopolitical conflicts [18]. Relatively little attention has been paid to unfriending in the context of public health. Although the COVID-19 pandemic has been and remains a highly politicized event [19,20], the role of social networks in the exchange of health-related information represents a unique and understudied context in which to consider unfriending behaviors and their potential impact on the function of health networks and information exchange. This paper draws from prior studies of politically motivated unfriending and the broader communication literature in an effort to better understand this phenomenon in the context of the COVID-19 pandemic. The subsequent subsections briefly summarize the information environment in the first year of the pandemic and the potential antecedents and consequences of unfriending behaviors. This is followed by a summary of the study’s guiding hypotheses.

### The COVID-19 Infodemic

Consistent with much of what we know about crisis communications [21-23], the acute emergence of the COVID-19 pandemic resulted in a fluid, ambiguous, and highly speculative information environment. Facilitated by the uncertainty of the emerging health crisis and the proliferation of nontraditional media outlets, the early days of the pandemic were marked by the rapid spread of misinformation, which often outpaced the ability of public health professionals to monitor and respond [24,25]. The early and ongoing politicization of public health responses to the COVID-19 pandemic further complicated the information environment by undermining the perceived legitimacy of public health messaging [20,26].

During this time, common misinformation themes ranged from genuine medical discrepancies—such as concerns that vaccines might contain live strains of the virus or impact fertility—to wild political conspiracies, including claims that vaccines contained 5G microchips or were designed to reduce the world’s population. In September 2020, the World Health Organization dubbed this phenomenon an “infodemic” and categorized it as a distinct public health crisis, running parallel and contributing to the viral pandemic itself. Early infodemic research highlighted both the extensive range of misinformation themes circulating on the web [27,28] and the role of homogeneous social networks in facilitating their spread [29]. Later research helped to clearly demonstrate the impact of these trends, revealing that exposure to misinformation led to increased vaccine hesitancy and decreased confidence in public health messaging [19,30].

Within this context, survey research showed that conversations and personal interactions during the COVID-19 pandemic had become increasingly stressful for health consumers, particularly in digital settings. Many reported strains in their personal and professional relationships owing to COVID-19 pandemic–related disagreements [12,13,31], and high levels of network filtration (ie, unfriending and selective avoidance) were observed among SNS users [15]. This study is primarily concerned with the motivations for and potential implications of these behaviors, which are discussed further in subsequent sections.

### The Antecedents and Consequences of Unfriending

In the context of social networks, unfriending represents a specific form of post hoc boundary regulation, whereby SNS users continually renegotiate their social interactions and informational exposure by breaking network ties with those who post unwanted or counterattitudinal content [32-34]. Notably, network curation and boundary regulation in web-based social networks can include a range of behaviors beyond unfriending—such as *following*, *blocking*, and *reposting*. Prior studies on social media use during the COVID-19 pandemic have considered the impacts of decisions such as which accounts or sources to follow for COVID-19 pandemic–related information [1]. This study—building on prior research in the fields of political science and communication [16,18,34]—focuses specifically on unfriending behaviors, which shape future information environments based on user reactions to information exposure.

From an academic standpoint, the salience of these behaviors arises from the potential motivations for unfriending. It has been argued that the reasons *why* SNS users unfriend others in their social networks may have significant implications in terms of their subsequent information exposure, beliefs, and behaviors. One line of inquiry has suggested that unfriending represents a form of selective avoidance, whereby SNS users engage in boundary regulation as a means of avoiding alternative viewpoints, thereby mitigating the cognitive dissonance that arises from exposure to counterattitudinal messaging [17,18,35,36]. Proponents of this theory warn that these behaviors represent a threat to public discourse insofar as they may homogenize information environments, creating echo chambers that are unreceptive to corrective information and vulnerable to radicalization [37,38].

It is worth emphasizing that selective avoidance may not, in and of itself, be a sufficient condition for the formation of web-based echo chambers. Indeed, the echo chamber hypothesis has arisen as a point of contention in recent years as political and communications scholars have debated both the theoretical and empirical merits of this argument. For example, Dubois and Blank [39] noted that modern information consumers operate in a “high-choice environment,” wherein processes of information seeking and learning are informed by a range of media options, thereby undercutting such concerns around web-based social networks. Bode [40] underscores this idea, noting that those who are most likely to engage in politically motivated unfriending on social media are typically more likely to encounter diverse political perspectives through other mediums. This is welcome news to those who place value on diverse, counterattitudinal information exposure. However, in each case, these observations apply to the most politically active and engaged SNS users, and the generalizability of this relationship to the context of health information remains unclear.

Although selective avoidance offers one potential motivation for unfriending, recent studies have suggested that unfriending may be a function of information utility rather than partisan predilections. For example, Neely [16] found a strong relationship between unfriending and SNS users’ perceptions of information credibility, wherein those who lacked confidence in the accuracy of information shared in their social network were substantially more likely to engage in unfriending. Metzger et al [41] reached similar conclusions, namely that selective exposure and avoidance appeared to be a function of how consumers assessed the credibility of an information source rather than the experience of any cognitive dissonance from being exposed to counterattitudinal information. These findings are consistent with the broader literature on media uses and gratifications, which identifies learning and information seeking among the most important determinants of media use and adoption, including in digital settings [42,43]. From this perspective, it could be argued that COVID-19 pandemic–related unfriending represents a form of boundary regulation driven by a desire to preserve the accuracy (and thus utility) of the user’s information environment.

With these considerations in mind, it is important to better understand users’ motivations for unfriending around the COVID-19 pandemic, as the circulation of accurate and reliable health information is essential for the effective management of public health crises. As public health policy in the United States becomes increasingly politicized, the need to understand these phenomena becomes more pressing. If SNS users engage in selective avoidance of pandemic-related information as a means of tuning out competing viewpoints, there may be a significant and systemic impact on the exchange of accurate and corrective health information. Namely, these individuals may be dissolving network connections that could prove to be a source of valuable mitigation, treatment, and vaccination information in the future. If, on the other hand, SNS users engage in unfriending as a means of safeguarding the accuracy and credibility of their informational environments (that is, breaking the network ties that spread health misinformation), then these behaviors may have a net positive effect on public health outcomes. It should be noted that this latter tendency is likely to be complicated by *hostile media effects* or the tendency of the partisan information consumers to interpret information credibility based on ideological predispositions [44,45]. This consideration is addressed further in the *Discussion* section below.

### Research Question and Hypotheses

Building on prior research, this study expanded the range of outcome measures typically used in studies of politically motivated unfriending to include 4 distinct categories of COVID-19 pandemic–related unfriending. These included unfriending in response to (1) posting about the COVID-19 pandemic too often, (2) posting information that was inconsistent with public health guidelines, (3) posting ideas or information about the COVID-19 pandemic that you disagree with, and (4) making political comments about the COVID-19 pandemic. Although there is likely to be some overlap between these categories in the reality of user experiences, they provide a more nuanced understanding of boundary regulation than the more general, binary measures of unfriending used in some prior studies. Given the dearth of research examining unfriending in a public health context, this study was undertaken in an exploratory spirit; however, 2 research questions and 3 directional hypotheses were considered when developing and conducting this research.

The overarching research questions guiding this analysis considered both the prevalence of Facebook unfriending during (and related) to the COVID-19 pandemic as well as the underlying motivations for engaging in COVID-19 pandemic–related boundary regulation:

1. Research question 1: *How prevalent (common) was COVID-19 pandemic–related unfriending on Facebook during the first year of the pandemic?*
2. Research question 2: *What factors motivated SNS users to engage in COVID-19 pandemic–related unfriending during the first year of the pandemic?*

First, it is hypothesized that utility motivations will predict unfriending in the case of posts that are inconsistent with public health guidance. In other words, those who rely heavily on Facebook as an important source of news and information about COVID-19 will be more likely to engage in boundary regulation (ie, unfriending) when confronted with information that they perceive to be inconsistent with public health guidance. This hypothesis is in line with both the fundamental premises of the uses and gratifications literature, as well as with prior research that has found perceptions of information credibility to be an important determinant of selective avoidance and unfriending [16,41].

Hypothesis 1: Unfriending in response to information that is “inconsistent with public health guidance” will be positively related to reliance on Facebook for news and information about COVID-19.

Prior research has also shown a consistent link between ideological intensity and politically motivated unfriending, wherein those with stronger ideological tendencies, regardless of political affiliation, are more likely to dissolve network ties in the case of political disagreement [18,34,40]. This hypothesis is consistent with the theory that selective avoidance mechanisms may motivate unfriending, as prior research has demonstrated a strong link between preferences for partisan media and ideological intensity [46,47].

Hypothesis 2: Unfriending in response to disagreement and politicization of the COVID-19 pandemic will be positively related to ideological intensity.

Finally, it is also hypothesized that COVID-19 pandemic–related unfriending—in the aggregate—will be positively related to a user’s number of Facebook friends. Prior research has suggested that SNS users are more likely to dissolve weak ties in the face of disagreement, and larger networks are believed to contain a greater number of weak-tie relationships [18,34,48]. This is a potentially problematic relationship because weak ties within a social network are believed to be essential for facilitating the exchange of diverse viewpoints and connecting users with corrective information sources across network clusters [14,49,50]. These ideas are addressed in the *Discussion* section below.

Hypothesis 3: COVID-19 pandemic–related unfriending will be positively related to the size of the user’s social network.

## Methods

### Overview

Situated in a larger study of web-based behavior during the COVID-19 pandemic, funding in support of this study was provided by the Florida Center for Cybersecurity (University of South Florida). The project began with a representative sample of 1000 American adults. The survey, fielded between January 9 and 12, 2021, used a stratified quota methodology and was collected through Prodege MR [51], an industry-leading market research provider. Quotas were determined using US Census data and balanced (by region of the country) to be representative based on age, sex, race, ethnicity, and education. The initial sample included 824 active Facebook users, which were used for the analysis summarized in the *Results* section below. As functionality (as it relates to network curation and boundary regulation) varies across SNS platforms, this study focuses on a single platform (Facebook) to avoid ambiguity and confusion as well as to ensure data validity. Facebook was chosen for this analysis, as it was the most commonly used social media platform (outside of YouTube) during the study period [52].

Survey participants with active Facebook accounts were asked whether they had unfriended someone on Facebook during the pandemic for each of 4 potential reasons. These included (1) posting about the COVID-19 pandemic too often, (2) posting information that was inconsistent with public health guidelines, (3) posting ideas or information about the COVID-19 pandemic that you disagree with, and (4) making political comments about the COVID-19 pandemic. Basic descriptive statistics were analyzed to determine the frequency of unfriending for each of the 4 potential reasons. Subsequently, a series of 4 logistic regression models were constructed to test the hypotheses outlined in the *Research Question and Hypotheses* section. The regression models were estimated as follows:

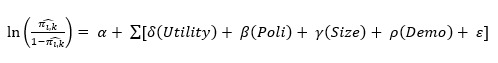

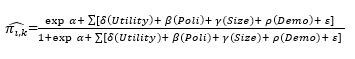





where 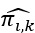 is the estimated probability that the *i*th case engaged in unfriending for the reason provided in category *k*; *Utility* is a vector of control variables measuring the user’s reliance on (and confidence in) Facebook as a source of COVID-19 pandemic–related news and information; *Poli* is a vector of political ideology controls; *Size* is a measure of the user’s social network size; and *Demo* is a vector of demographic controls. The *Poli* vector contains 2 variables measuring party affiliation and ideological intensity. The *Utility* vector includes three questions measuring (1) reliance on social media to learn about COVID-19, (2) frequency of COVID-19 information engagement on social media, and (3) confidence in the accuracy of COVID-19 pandemic–related information on social media. Table 1 summarizes the control variables for the sample, including descriptive statistics and measurement or coding rules.

**Table 1:** Variable coding and descriptive statistics (n=824).

Variables			Coding		Values
Facebook friends, mean (SD)			Continuous; range=0-134,000 (log-transformed)		571.9 (5096.7)
**Reliance on Facebook for COVID-19 pandemic–related information, n (%)**					
	Not at all	Reference category		161 (19.5)	
	A little	1=yes; 0=no		258 (31.3)	
	A lot	1=yes; 0=no		221 (26.8)	
	A great deal	1=yes; 0=no		184 (22.3)	
**Frequency of reading about COVID-19 pandemic–related information, n (%)**					
	Less often	Reference category		207 (25.1)	
	Once a week	1=yes; 0=no		92 (11.2)	
	A few days a week	1=yes; 0=no		237 (28.8)	
	Every day	1=yes; 0=no		288 (35)	
**Confident in accuracy of information on Facebook, n (%)**					
	Neither agree nor disagree	Reference category		202 (24.5)	
	Strongly agree	1=yes; 0=no		65 (7.9)	
	Somewhat agree	1=yes; 0=no		214 (26)	
	Somewhat disagree	1=yes; 0=no		162 (19.7)	
	Strongly disagree	1=yes; 0=no		181 (22)	
**Party affiliation, n (%)**					
	Democrat	Reference category		310 (37.6)	
	Independent	1=yes; 0=no		195 (23.7)	
	Republican	1=yes; 0=no		205 (24.9)	
	Nonvoter	1=yes; 0=no		114 (13.8)	
**Ideological intensity, n (%)**					
	None	Reference category		323 (39.2)	
	Low	1=yes; 0=no		302 (36.7)	
	High	1=yes; 0=no		199 (24.2)	
Sex, n (%)			1=female; 0=male		439 (53.3)
Age (years), mean (SD)			Continuous; range=18-86 (log-transformed)		47.6 (16.4)
College education, n (%)			1=college degree or higher		286 (34.7)

For this analysis, *ideological intensity* was determined by asking respondents to describe their political ideology from among the following options: (1) very liberal, (2) somewhat liberal, (3) moderate, (4) somewhat conservative, and (5) very conservative. The *very liberal* and *very conservative* responses were recoded as *high intensity*, whereas *somewhat liberal* and *somewhat conservative* were recoded as *low intensity*. *Moderate* was recorded as *none*. To measure network size, respondents were asked to self-report their current number of Facebook friends, and this variable was log-transformed for the purposes of analysis.

For the *Utility* variable, reliance on Facebook was measured by asking respondents: *How much have you relied on social media to stay informed about the COVID-19 pandemic?* Response options included (1) a great deal, (2) a lot, (3) a little, and (4) not at all. The frequency of COVID-19 pandemic–related information engagement was measured by asking respondents: *On average, how often do you read information about COVID-19 on social media?* Response options included (1) every day, (2) a few days a week, (3) once a week, and (4) less often. Confidence in the accuracy of COVID-19 pandemic–related information was measured by asking respondents to rate their agreement with the following statement: *I am confident in the accuracy of the information I see about COVID-19 on social media*. Response options included a 5-point Likert scale ranging from *strongly agree* to *strongly disagree*.

Finally, demographic control variables were included for sex, age, and college education. For sex, male was omitted as the reference category. Education was recoded as a binary variable, with *less than college degree* omitted as the reference category. Age was measured as a continuous variable and log-transformed for this analysis. Additional demographic measures for race and ethnicity were collected but were excluded from this analysis owing to their multicollinearity with party affiliation.

### Ethical Considerations

The methodology used in this study has been classified as “exempt from IRB review” by the University of South Florida’s institutional review board. This determination was made by the institutional review board for the initial phase of this project (STUDY #000078) because the survey was conducted through a third-party panel vendor and the research team did not interact directly with participants. Furthermore, no personally identifying information was collected by or transferred to the researchers. Although the third-party panel vendor collects these data, only deidentified, secondary data are transmitted to the researchers.

## Results

Table 2 provides a summary of the responses for each of the 4 unfriending categories. In total, 31.7% (261/824) of the Facebook users reported at least 1 type of COVID-19 pandemic–related unfriending during the first year of the pandemic. This is consistent with the levels of unfriending observed in other recent studies of US-based SNS users [16,53]. Making political comments about the COVID-19 pandemic was the most commonly cited reason for COVID-19 pandemic–related unfriending, with approximately a quarter of respondents (191/824, 23.2%) indicating that they had done so. Approximately 1 in 5 (162/824, 19.7%) users reported unfriending members of their social network for posting information that was inconsistent with public health guidelines, whereas 17.1% (141/824) of users did so when users posted COVID-19 pandemic–related information that they disagreed with. Posting about the COVID-19 pandemic too often was the least common reason for unfriending, which is unsurprising given the ubiquity of pandemic-related content during this time. A correlational analysis showed that it was common for respondents who engaged in COVID-19 pandemic–related unfriending to unfriend others for multiple reasons.

To better understand the antecedents of these boundary-regulating behaviors, 4 binary logit models were constructed to examine each unfriending category individually. For the purposes of this discussion, the results are presented as odds ratios (e^b^), which are easier to interpret than traditional β coefficients [54] as they represent changes in the odds of unfriending based on a 1-unit increase in the independent or control variable, *ceteris paribus*. Odds ratios >1 indicate an increase in the odds of a given response, whereas ratios <1 indicate a decrease in the odds. When the odds ratios are <1, they can be converted for comparison to positive values (ie, 1/e^b^). The results are discussed in Table 3 with a particular emphasis on the hypotheses of the study.

**Figure 1:**
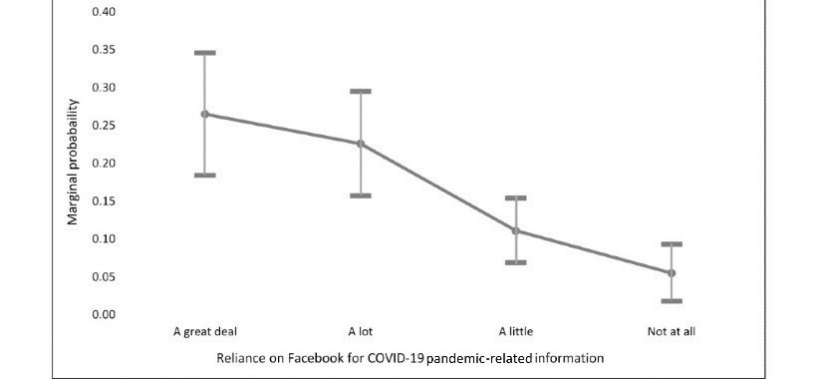
Marginal effects of reliance on Pr(Unfriending).

Table 3 summarizes models 1 and 2, which examine unfriending in response to “posting about COVID-19 too often” and “posting content that was inconsistent with public health guidance,” respectively. Hypothesis 1 posited that unfriending in response to information that is “inconsistent with public health guidance” will be positively related to reliance on Facebook for news and information about COVID-19. The data supported this hypothesis, as those who relied on Facebook *a great deal* to learn and stay informed about the COVID-19 pandemic were over 6 times more likely to have unfriended for this reason (model 2, e^b^=6.171). Across each categorical response, as reliance on Facebook for COVID-19 pandemic–related information increased, so did the likelihood of unfriending in response to information that contradicted public health guidance. Figure 1 depicts the marginal increase in the likelihood of this type of unfriending across varying levels of reliance on Facebook, *ceteris paribus*. The probability of unfriending among those who did not rely on Facebook for COVID-19 pandemic–related information was 0.05 but increased consistently to 0.26 among those who relied on Facebook *a great deal*.

**Table 2:** Frequency of COVID-19 pandemic–related unfriending on Facebook (n=824).

Since the start of the pandemic, have you “unfriended” someone on Facebook for any of the following reasons?	Yes, n (%)
Posting about COVID-19 too often	114 (13.8)
Posting information that was inconsistent with public health guidelines	162 (19.7)
Posting ideas or information about COVID-19 that you disagree with	141 (17.1)
Making political comments about COVID-19	191 (23.2)

**Table 3:** Logistic regression model 1 (posting about the COVID-19 pandemic too often) and model 2 (posting information that was inconsistent with public health guidelines).

		Model 1, odds ratio (95% CI; SE)	Model 2, odds ratio (95% CI; SE)
Facebook friends (ln)^a^		1.109 (0.954-1.289; 0.085)	1.069 (0.934-1.223; 0.736)
**Reliance on Facebook (COVID-19 pandemic–related information)**			
	Not at all	—^b^	—
	A little	0.998^c^ (0.361-2.757; 0.517)	2.145^c^ (0.999-4.605; 0.836)
	A lot	3.769^c^ (1.189-11.951; 2.219)	4.997^c^ (2.083-11.987; 2.231)
	A great deal	4.255 (1.132-13.717; 2.541)	6.171^c^ (2.541-14.986; 2.793)
**Frequency of COVID-19 social media engagement**			
	Less often	—	—
	Once a week	0.424 (0.094-1.911; 0.325)	0.353 (0.137-0.911; 0.171)
	A few days a week	0.893 (0.335-2.379; 0.446)	0.679 (0.356-11.987; 0.224)
	Every day	0.825 (0.296-2.299; 0.431)	0.847 (0.431-1.665; 0.292)
**Confident in the accuracy of COVID-19 pandemic–related information**			
	Neither agree nor disagree	—	—
	Strongly agree	3.553 (1.619-7.797; 1.425)	1.837 (0.874-3.861; 0.697)
	Somewhat agree	1.349^c^ (0.689-2.642; 0.462)	0.852 (0.484-1.499; 0.246)
	Somewhat disagree	1.542 (0.722-3.294; 0.597)	1.587 (0.864-2.917; 0.493)
	Strongly disagree	1.951 (0.797-4.777; 0.891)	1.296 (0.650-2.587; 0.457)
**Party affiliation**			
	Democrat	—	—
	Independent	1.028 (0.566-1.867; 0.313)	0.729 (0.427-1.242; 0.198)
	Republican	1.283 (0.723-2.277; 0.376)	0.479^c^ (0.286-0.799; 0.125)
	Nonvoter	0.541 (0.249-1.172; 0.213)	0.434^c^ (0.213-0.883; 0.157)
**Ideological intensity**			
	None	—	—
	Low	0.928 (0.532-1.618; 0.263)	1.603^d^ (0.988-2.600; 0.396)
	High	1.592^d^ (0.929-2.728; 0.437)	2.343^c^ (1.401-3.918; 0.615)
Sex (female)		0.508^c^ (0.322-0.802; 0.118)	0.602^c^ (0.408-0.887; 0.119)
Age (years; ln)^a^		0.269^c^ (0.146-0.497; 0.084)	0.372^c^ (0.213-0.651; 0.106)
College education (yes)		1.892^c^ (1.190-3.008; 0.448)	2.107 (1.393-3.187; 0.445)
Constant		3.812 (0.292-49.760; 4.997)	2.068 (0.184-23.229; 2.552)
−2 Log likelihood		−266.709 (N/A^e^)	−332.373 (N/A)
Pseudo *R*^2^		0.188 (N/A)	0.173 (N/A)

^a^The variable was log-transformed.

^b^Reference categories.

^c^*P*≤.05.

^d^*P*≤.10.

^e^N/A: not applicable.

Republicans were 2 times less likely to have unfriended others in response to information that contradicted public health guidance (ie, 1/e^b^ or 1/0.479=2.09). This is consistent with the politicization of the COVID-19 pandemic and particularly the observation that Republican voters have been less likely to express confidence in public health guidance. The politicization of public health policy is also highlighted here by the fact that the likelihood of unfriending in response to information that contradicted public health guidelines was higher among those with greater ideological intensity (ie, those with high ideological intensity were 2 times more likely to have unfriended than self-reported moderates).

**Figure 2:**
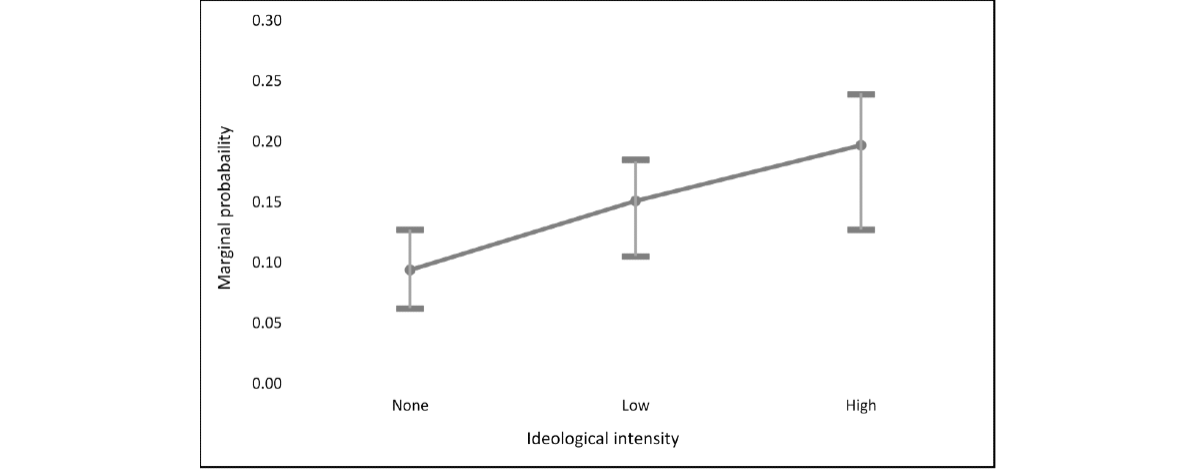
Marginal effects of ideological intensity on Pr(Unfriending).

Table 4 summarizes models 3 and 4, which examine unfriending in response to “posting ideas or information about COVID-19 that you disagree with” and “making political comments about COVID-19,” respectively. For hypothesis 2, the results showed strong support. Those with high ideological intensity were >2 times as likely to unfriend someone in response to disagreement over COVID-19 pandemic–related information and 1.7 times more likely to unfriend someone who made political comments about the COVID-19 pandemic. Specifically, Republicans were less likely to have unfriended in response to disagreement, but there were no significant differences in party affiliation when it came to unfriending over political comments. Figure 2 shows marginal increases in the likelihood of unfriending over disagreement across varying levels of ideological intensity, *ceteris paribus*. The probability of unfriending in response to a COVID-19 pandemic–related disagreement was 0.09 among those with no ideological intensity and increased consistently to 0.18 for those with high ideological intensity.

It is important to note that reliance on Facebook for news and information about the COVID-19 pandemic was also a significant predictor of unfriending in both models 3 and 4. This may potentially suggest that those who were more reliant on Facebook as a source of pandemic-related information had less patience for politicization of the pandemic and were more likely to remove sources of politicization out of a utility motivation.

Age and education were significant predictors of unfriending across all 4 models. In each case, the likelihood of unfriending decreased as age increased, which may reflect differences in platform literacy among other possible factors [34]. In each case, college-educated respondents were significantly more likely to engage in COVID-19 pandemic–related unfriending. This could also reflect differences in platform literacy, although it may also be a function of higher levels of confidence in public health guidance, thus suggesting a potential utility motivation for unfriending. Additional research would be needed to further examine these speculations.

Finally, hypothesis 3 posited that *COVID-19 pandemic–related unfriending will be positively related to the size of the user’s social network.* This was only confirmed in the case of “making political comments” about the COVID-19 pandemic. Although the magnitude of this effect is not as substantial as that seen in some prior studies of politically motivated unfriending [18,34], it does suggest (inferentially) a greater tendency to dissolve weak-tie relationships in the face of unwanted politicization.

**Table 4:** Logistic regression model 3 (posting ideas or information about the COVID-19 pandemic that you disagree with) and model 4 (making political comments about the COVID-19 pandemic).

			Model 3, odds ratio (95% CI; SE)		Model 4, odds ratio (95% CI; SE)
Facebook friends (ln)^a^			1.078 (0.936-1.240; 0.077)		1.186^b^ (1.049-1.339; 0.074)
**Reliance on Facebook (COVID-19 pandemic–related information)**					
	Not at all (reference category)	—^c^		—	
	A little	2.268^b^ (1.018-5.051; 0.927)		1.484 (0.797-2.762; 0.470)	
	A lot	4.827^b^ (1.950-11.946; 2.232)		4.735^b^ (2.171-10.327; 1.884)	
	A great deal	6.314^b^ (2.497-15.964; 2.988)		4.554^b^ (2.494-12.372; 2.269)	
**Frequency of COVID-19 social media engagement**					
	Less often (reference category)	—		—	
	Once a week	0.559 (0.223-1.406; 0.263)		0.722 (0.347-1.502; 0.269)	
	A few days a week	0.728 (0.353-1.500; 0.269)		0.677 (0.370-1.239; 0.209)	
	Every day	0.777 (0.373-1.616; 0.290)		0.696 (0.372-1.301; 0.222)	
**Confident in accuracy of COVID-19 pandemic–related information**					
	Neither agree nor disagree (reference category)	—		—	
	Strongly agree	1.984^d^ (0.962-4.093; 0.733)		2.063^b^ (1.034-4.116; 0.727)	
	Somewhat agree	0.812 (0.450-1.462; 0.244)		1.110 (0.651-1.891; 0.302)	
	Somewhat disagree	1.404 (0.748-2.635; 0.451)		1.563 (0.882-2.771; 0.456)	
	Strongly disagree	1.224 (0.604-2.479; 0.441)		2.703^b^ (1.464-4.989; 0.845)	
**Party affiliation**					
	Democrat (reference category)	—		—	
	Independent	0.765 (0.436-1.339; 0.219)		1.107 (0.685-1.788; 0.271)	
	Republican	0.501^b^ (0.295-0.852; 0.136)		0.827 (0.524-1.306; 0.192)	
	Nonvoter	0.603 (0.301-1.209; 0.214)		0.635 (0.336-1.199; 0.206)	
**Ideological intensity**					
	None	—		—	
	Low	1.703^b^ (1.034-2.803; 0.433)		1.581^b^ (1.023-2.444; 0.351)	
	High	2.160^b^ (1.287-3.625; 0.571)		1.706^b^ (1.062-2.740; 0.412)	
Sex (female)			0.729 (0.484-1.098; 0.152)		0.932 (0.656-1.325; 0.167)
Age (years; ln)^a^			0.407^b^ (0.228-0.726; 0.120)		0.589^b^ (0.354-0.979; 0.153)
College education (yes)			1.954^b^ (1.287-2.964; 0.415)		1.541^b^ (1.058-2.246; 0.296)
Constant			1.076^b^ (0.084-13.781; 1.399)		0.212 (0.022-2.005; 0.243)
−2 Log likelihood			−320.174 (N/A^e^)		−393.548 (N/A)
Pseudo *R*^2^			0.143 (N/A)		0.108 (N/A)

^a^The variable was log-transformed.

^b^*P*≤.05.

^c^Reference categories.

^d^*P*≤.10.

^e^N/A: not applicable.

## Discussion

### Overview

This study examined Facebook unfriending during and related to the first year of the COVID-19 pandemic. Consistent with recent research [16,41], the results suggest that boundary regulation through unfriending is a function of both information utility concerns and partisan impulses, although the magnitude of the effects associated with utility appears to be greater. Several important conclusions can be drawn from these findings.

First, although many prior studies on unfriending have focused on partisan or political motivations, the results of this study underscore the importance of utility motivations in understanding boundary regulation and unfriending. Among the predictor variables considered in this analysis, reliance on Facebook for pandemic-related information was the most substantial predictor of unfriending in each instance, particularly when it came to content that contradicted public health guidance. As noted in the *Research Question and Hypotheses* section, the uses and gratifications literature tells us that information seeking and learning are primary motivators of media adoption [42,43], and thus it makes sense that those who view Facebook and other social media platforms as sources of *news*—rather than merely as social spaces—would be more likely to engage in boundary-regulating efforts to ensure the accuracy, reliability, and utility of their future information exposure.

This finding, which is consistent with that of other recent studies [16,41], helps to enrich and contextualize our understanding of unfriending behavior. Although some have cautioned that the customizability of SNSs could lead to partisan filtration and homogenization [37,38], there appear to be more nuanced motivations at work in how SNS users construct and maintain their social networks. Specifically, SNS users who rely on social media for health-related news and information appear to be more, if not primarily, concerned with ensuring an accurate and reliable information environment than with muting opposing viewpoints. Although superficially this may be an optimistic interpretation of the findings, it should be tempered by our understanding of *hostile media effects*, which remind us that information consumers are often inclined to interpret the truth and accuracy of information through the lens of their existing ideological tendencies [44,45]. To the extent that this is true in the public health context, those who engage in unfriending out of even purely utilitarian motives may still be inadvertently limiting their subsequent exposure to important and potentially corrective information. At the least, this consideration warrants further research and examination.

Although the results suggest that utility motives might be the most compelling antecedent of unfriending behavior, there is still evidence of significant partisan effects at play in COVID-19 pandemic–related unfriending. Notably, the results show that those with high partisan intensity are more likely to engage in unfriending under nearly all circumstances, and particularly in the face of disagreement. The intense politicization of the COVID-19 pandemic [19,20,55] is likely to contribute to this finding, which is also consistent with prior studies of politically motivated unfriending [18,34,40]. Indeed, the results fall out in a pattern consistent with what we know of public opinion and pandemic-related policies. For instance, Republicans were significantly less likely than Democrats to have unfriended in response to information that was inconsistent with public health guidelines, which is unsurprising given the lower levels of confidence in public health guidance and pandemic mitigation measures exhibited by Republican voters throughout the pandemic [55,56]. Over time, these observed patterns of unfriending could lead to 2 distinct web-based information environments based on political affiliation and ideology.

Arguably, as SNS users become increasingly reliant on platforms such as Facebook for news and information, it is possible that partisan motivations for boundary regulation may become even stronger, particularly among those with high ideological intensity. As technological advances have led to a proliferation of media options, research has shown a growing tendency toward confirmation bias and selective exposure among American consumers [36]. Given the fact that intense partisans exhibit a greater tendency to favor congenial media sources [47,57], it is reasonable to suspect that this may be reflected in boundary-regulating behaviors such as unfriending over time. On the one hand, it has been suggested that these tendencies are unlikely to result in partisan echo chambers in any strict sense of the word, as the high-choice nature of the media environment means that users in homogenized social networks are still likely to encounter counterattitudinal information through “diverse media diets” [39]. Others have suggested that those high-intensity partisans who are most likely to engage in politically motivated unfriending are also more likely to encounter diverse opinions through various media sources [40].

However, there are invariably downsides to such behavior, regardless of whether consumers maintain other forms of exposure to counterattitudinal information. For example, Stroud [47] found that partisan selective exposure is related to increased polarization, which in this case could further entrench the politicization of public health discourse. There is also evidence that misinformation is more likely to circulate in homogenized web-based networks that have undergone these processes of filtration and ideological boundary regulation [29]. In the context of public health, exposure to misinformation has been linked to undesirable health outcomes and behaviors. For example, both Chen et al [30] and Neely et al [19] found a significant link between exposure to COVID-19 pandemic misinformation and vaccine hesitancy as well as decreased confidence in public health guidance. As consumers increasingly rely on SNSs for health information and learning, boundary regulation motivated by partisan preferences could potentially increase the likelihood of misinformation exposure and decrease the frequency of exposure to corrective information.

Finally, hypothesis 3 proposed that COVID-19 pandemic–related unfriending would be most common among those with larger social networks. Overall, the results did not support this hypothesis. Although those with larger Facebook networks were more likely to engage in each type of unfriending, this relationship was only statistically significant in the case of “making political comments about the COVID-19 pandemic.”

Prior research on politically motivated unfriending has suggested that SNS users are more likely to break weak-tie relationships than strong-tie relationships such as those between close friends and family members. In the context of the COVID-19 pandemic, this appears to be true in the face of *politicization* (ie, making political comments about the COVID-19 pandemic). This makes sense in light of the current findings, that is, those who are concerned with protecting the informational integrity and credibility of their social networks will be less tolerant of politicization in that information environment, particularly when it originates from those with whom they are less closely connected.

Therefore, there are some concerns to consider with regard to this finding. It has been argued that weak ties are more likely to fill *brokerage* roles in social networks and therefore play an important part in promoting exposure to diverse viewpoints and corrective information. Granovetter [14] notes that “...those to whom we are weakly tied are more likely to move in circles different from our own and will thus have access to information different from that which we receive.” An extensive body of literature has affirmed this hypothesis [50,57], and thus, tendencies to unfriend weak ties could lead to more homogenized information environments, which might further limit SNS users’ exposure to accurate and corrective health information. The implications of this tendency for public health learning on social networks require further consideration.

From a practical perspective, the findings outlined above suggest that health practitioners and public health officials should consider the factors underlying network curation and boundary regulation when engaging with health content in digital spaces. The results suggest that many SNS users deliberately regulate the boundaries of their social networks in an effort to ensure informational credibility and accuracy. However, prior research has also suggested that many SNS users do not follow or engage with authoritative medical or scientific sources on social media [1]. A greater emphasis on platform literacy and social media capacity may help public health organizations to gain visibility in digital spaces and increase their influence as authoritative information sources in modern public health discourse. Among other steps, this may include a more deliberate focus on institutional policies surrounding social media outreach and engagement [58].

Furthermore, although health practitioners and public health organizations focus primarily on communicating the science of public health, it is increasingly necessary to acknowledge the widespread and pernicious effects of politicization in this arena [20]. Although the results of this study suggest that information utility may be a more potent driver of boundary regulation, there is still evidence that some SNS users deploy tools such as unfriending to filter out opposing points of view. Over time, these behaviors can lead to the formation of negative feedback loops that reinforce errant beliefs and amplify misinformation. It is increasingly necessary for health professionals to intentionally communicate across ideological communities and for health care providers to be armed with the tools and information needed to empathetically address patient concerns that arise from politicized health information. Leveraging partnerships with respected thought leaders within political and ideological circles may be a viable means of helping to overcome these challenges.

### Limitations

Although this study shows that COVID-19 pandemic–related unfriending has been a function of both utility-based motivations and partisan predilections, the larger effect of social networks and boundary regulation on public health outcomes still requires considerable examination. Specifically, we need a deeper understanding of how SNS users frame and adjudicate the reliability of health-related information that they encounter on the web and how this relates to their boundary-regulating behaviors. Users who relied the most on Facebook for information about the COVID-19 pandemic were more likely to unfriend those who posted information that was inconsistent with public health guidelines, but our understanding of this relationship is limited by untested assumptions about users’ understanding of public health guidelines. Among other considerations, a better understanding of how SNS users rate the strength of network ties, particularly among those whom they unfriend, would help to deepen our understanding of boundary regulation and its potential impact on information exposure and public health outcomes.

These results are also limited by our lack of specificity regarding the types of content that prompt unfriending behaviors. A more nuanced mixed methods analysis might help to deepen our understanding and further contextualize the current findings. Finally, this study focused specifically on Facebook as the most widely used SNS platform in the United States [52]. Although focusing on a specific platform helps to ensure data validity, it is worth emphasizing that patterns of use and platform attributes may result in significant differences in boundary regulation when compared with other social media platforms. Moving forward, it is important to consider whether and to what extent these findings are consistent across other widely used platforms (such as Twitter and Instagram).

## Abbreviations

SNS: social networking site
